# Alteration of taste perception, food neophobia and oral microbiota composition in children with food allergy

**DOI:** 10.1038/s41598-023-34113-y

**Published:** 2023-04-28

**Authors:** Enza D’Auria, Camilla Cattaneo, Simona Panelli, Carlotta Pozzi, Miriam Acunzo, Stella Papaleo, Francesco Comandatore, Chiara Mameli, Claudio Bandi, Gianvincenzo Zuccotti, Ella Pagliarini

**Affiliations:** 1 Department of Pediatrics, Buzzi Children’s Hospital, University of Milan, 20154 Milan, Italy; 2 Sensory & Consumer Science Lab (SCS_Lab), Department of Food, Environmental and Nutritional Sciences, University of Milan, 20133 Milan, Italy; 3 Pediatric Clinical Research Center “Invernizzi”, Department of Biomedical and Clinical Sciences, University of Milan, 20157 Milan, Italy; 4 Pediatric Clinical Research Center “Invernizzi”, Department of Biosciences, University of Milan, 20157 Milan, Italy

**Keywords:** Microbiome, Paediatric research, Physiology

## Abstract

Currently, the mechanisms underlying sensory perception and sensory performance in children with food allergies are far from being understood. As well, only recently, single research afforded the oral host-commensal milieu, addressing oral microbial communities in children with peanut allergies. To bridge the current gaps in knowledge both in the sensory and microbial fields, a psychophysiological case–control study was performed in allergic children (n = 29) and a healthy sex-age-matched control group (n = 30). Taste perception, food neophobia, and liking were compared in allergic and non-allergic children. The same subjects were characterized for their oral microbiota composition by addressing saliva to assess whether specific profiles were associated with the loss of oral tolerance in children with food allergies. Our study evidenced an impaired ability to correctly identify taste qualities in the allergic group compared to controls. These results were also consistent with anatomical data related to the fungiform papillae on the tongue, which are lower in number in the allergic group. Furthermore, distinct oral microbial profiles were associated with allergic disease, with significant down-representations of the phylum *Firmicutes* and of the genera *Veillonella* spp., *Streptococcus* spp., *Prevotella* spp., and *Neisseria* spp. For the first time, this study emphasizes the link between sensory perception and food allergy, which is a novel and whole-organism view of this pathology. Our data indicated that an impaired taste perception, as regards both functionality and physiologically, was associated with food allergy, which marginally influences the food neophobia attitude. It is also accompanied by compositional shifts in oral microbiota, which is, in turn, another actor of this complex interplay and is deeply interconnected with mucosal immunity. This multidisciplinary research will likely open exciting new approaches to therapeutic interventions.

## Introduction

Food allergy (FA) is an inappropriate immune system reaction towards food or its component^1^. FA includes different disorders with symptoms ranging from most severe reactions, such as anaphylaxis, to respiratory, cutaneous, and/or gastrointestinal symptoms^2^. In the last 100 years, FA prevalence has risen in western countries^3^, ranging from 1 to 10%, to different food allergens and in other populations and age groups^4^. This rise in FA, and in general in allergic disease, has prompted theorizations that led to the development of concepts such as the “hygiene hypothesis”, according to which such rise, and the impairment of tolerogenic immune functions, would be linked to improved sanitization practices and, in general, to changes in lifestyle that impacted interactions of the human host with external microbes, as well as with its commensal microbiota^5,6^.

From a therapeutic point of view, the mainstay of FA treatment is food avoidance and emergency treatments in the event of accidental ingestion. This poses significant physical, economic, and social burdens^2^. In addition, due to the need to avoid specific food groups, allergic toddlers and children have to acquire or adapt to different food preferences^7,8^, with an impact on their taste and sensory perception. For instance, infants and children diagnosed with cow’s milk allergy from an early age, and fed with casein-hydrolyzed formulas, are reported to be more willing to consume later-in-life foods characterized by bitter, sour, and savory tastes^7,9^. Another critical point is that the specific aversion toward the offending food might lead children suffering from one or more FA to a decreased interest in food^10^, which could make it a barrier to maintaining a varied dietary behavior^11^. Indeed, neophobic traits seem to characterize young children with FA^12^, and children and pre-adolescents who have outgrown their allergies^13^. This would explain why previously diagnosed allergic children and adolescents continue not consuming the offending food even after resolving their allergy, often because they reported that foods have a horrible and strange taste^14,15^. It is still not clear whether this phenomenon may be accentuated or not by biological mechanisms related to the gustatory system, which operates during and after ingestion, detecting and monitoring nutrients and potentially noxious compounds present in food^16^. As a general rule, the different reactivity of taste receptor mechanisms for nutrients and harmful molecules makes the sensory pathways more sensitive (i.e., lower threshold) in detecting the latter compared to nutrients. The low threshold (and low desensitization) for harmful molecules may decrease certain foods’ consumption. In contrast, the early choices and preferences of other food types may be promoted by nutrient sensors’ high threshold (and strong desensitization)^17,18^. However, research in the sensory field with a specific focus on allergic diseases and FA is still in its infancy. There is a lack of work on the mechanisms underlying sensory perception and sensory performance (i.e., gustatory and olfactory abilities) in children with FA. Most of the published research, which is not extensive, focused on toddlers’ liking and preferences for artificial milk substitutes and how they later affect flavor likes and dislikes (e.g.,^7,10,18^). Thus, specific psychophysiological studies addressing sensory perception, rather than just liking or preferences, are warranted. The assumption that children’s disease status may be linked to reduced chemosensory perceptions has been previously supported (for a review, see^18^), and taste impairments have been associated with numerous pathologies, such as cardiovascular disease, obesity, and diabetes^19–21^.

Another important key to FA is the role of the microbiota. Currently, as also mentioned above, growing evidence links the increasing prevalence of FA with a dysregulated homeostatic interaction between the host and its commensal bacterial communities, with microbial imbalances and dysbiosis preceding the onset of food sensitization and playing crucial roles in achieving or not oral tolerance^5,6,22–24^. Microbial communities play a crucial role in early host immunological development^25^ and affect food tolerance in several ways, e.g., via the microbial metabolites secretion (e.g., short-chain fatty acids, SCFA, with anti-inflammatory activities and involved in epigenetic regulation of the immune system), and the expression of specific cellular components^26^. Moreover, differences in oral microbial communities are reportedly linked to interindividual differences in taste perception^27–30^.

Disruption in the balanced network of interactions between the host and its microbes results in a T-helper 2 (Th2)-biased immune response when reacting to innocuous antigens as food^31^. Most current data in the literature focused on the structure of gut microbiota. To the best of our knowledge, only a very recent paper affords the oral host-commensal milieu, addressing oral microbial communities in food allergy^32^.

However, the oral cavity is the first meeting place between most antigens and the immune system, with antigen exposure and presentation by antigen-presenting cells. In homeostatic conditions of the oral cavity, the immune system keeps effective surveillance, tolerating commensal microorganisms, avoiding inflammatory responses, and modulating immune tolerance to antigens^33^.

With these premises, to bridge the current gaps in knowledge both in the sensory and microbial field, a psychophysiological case–control study focusing on taste perception (i.e., ability to recognize taste and number of fungiform papillae), food neophobia, and food liking was performed in allergic children and healthy sex-age matched control group. The same subjects were also characterized for their oral microbiota composition by addressing saliva to assess whether specific profiles were associated with food allergy.

## Results

### Patients’ characteristics

Overall, we collected data from 59 children, of which 29 were allergic and 30 were non-allergic. In the allergic group, 14 (48.3%) were girls and 15 (51.7%) boys, with a mean age of 10.5 ± 2.8 years. In the control group, 14 children (46.7%) were girls and 16 (53.3%) boys, whilst the mean age was 10.1 ± 1.6 years. No differences in terms of gender (χ^2^ = 0.02, p = 0.9) or age distribution (t = − 0.76, p = 0.4) between the allergic and control groups were observed.

The general and clinical features of allergic and control subjects are shown in Table 1.

**Table 1:** General and clinical features of cases and controls.

Characteristics	Allergic (ALL) n = 29	Controls (C) n = 30
Gender (F:M)	14:15	14:16
Age (mean ± SD)	10.7 ± 2.8	10.1 ± 1.6
Food neophobia level (n, %)		
Neophilics	14 (48%)	18 (60%)
Neophobics	15 (52%)	12 (40%)
Food allergy		
Tree nuts/peanuts, n (%)	20 (69%)	
Cow’s milk, n (%)	4 (14%)	
Fish, n (%)	2 (7%)	
Eggs, n (%)	1 (3%)	
Wheat, n (%)	1 (3%)	
Other foods, n (%)	1 (3%)	

### Taste sensitivity

**Figure 1:**
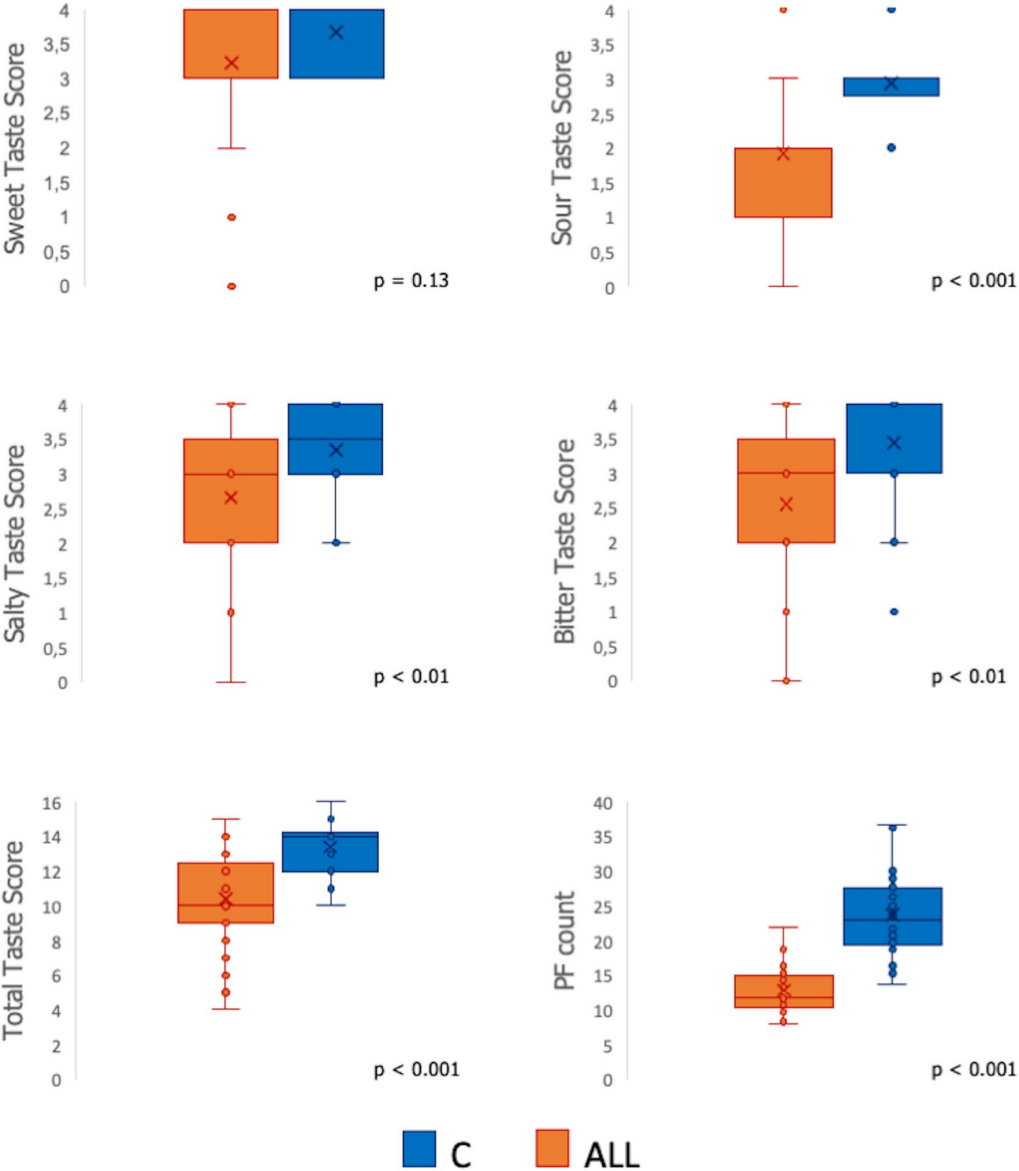
Boxplots showing the differences on both global (TTS) and single scores (Sweet Taste Score, Sour Taste Score, Salty Taste Score and Bitter Taste Score) as a function of allergic condition (ALL: Allergic group; C: Control group). The plots provide a representation of data distribution, individual raw observations, the median (horizontal line) ± IQR (box) within each group. Only statistically significant pairwise differences observed after post hoc Dunn’s test with Bonferroni correction are presented (**p < 0.01; ***p < 0.001).

To explore whether allergic conditions dealt differently with gustatory abilities, differences in both single taste scores (Sweet Taste Score, Sour Taste Score, Salty Taste Score, and Bitter Taste Score) and Total Taste Score (TTS) between allergic and controls separate Mann–Whitney U tests were performed. Raw scores, distributions, and significant comparisons between allergic and controls for both TTS, single taste scores, and Fungiform Papillae (FP) counts are displayed in Fig. 1.

Overall, the main effect of allergic condition on single taste scores was observed, with allergic subjects showing a lower recognition ability for Sour Taste Score (Mann–Whitney U value = 168, p < 0.001), Salty Taste Score (Mann–Whitney U value = 282, p < 0.01) and Bitter Taste Score (Mann–Whitney U value = 238, p < 0.01). These difficulties of being allergic in correctly identifying the different taste qualities resulted in considerably worse global taste performances (TTS) compared to controls (Mann–Whitney U value = 141.5, p < 0.001). The main effect of allergic conditions on FP count was also detected (t = 8.91, p < 0.001), with controls showing a greater FP density than allergic children.

Considering sex-related differences within each group, according to the Mann–Whitney test, no significant differences (p > 0.05) in gustatory abilities were found.

### Children’s food neophobia, liking, and taste sensitivity

To assess whether Group-related (allergic *vs* controls) and sex-related differences were associated with Food Neophobia, a 2-way ANOVA was performed.

Food neophobia attitudes appeared to be marginally influenced by the allergic condition (p = 0.10), showing a higher neophobic attitude for allergic than controls. No significant difference between boys and girls was found regarding the food neophobia score, nor for the interaction.

**Figure 2:**
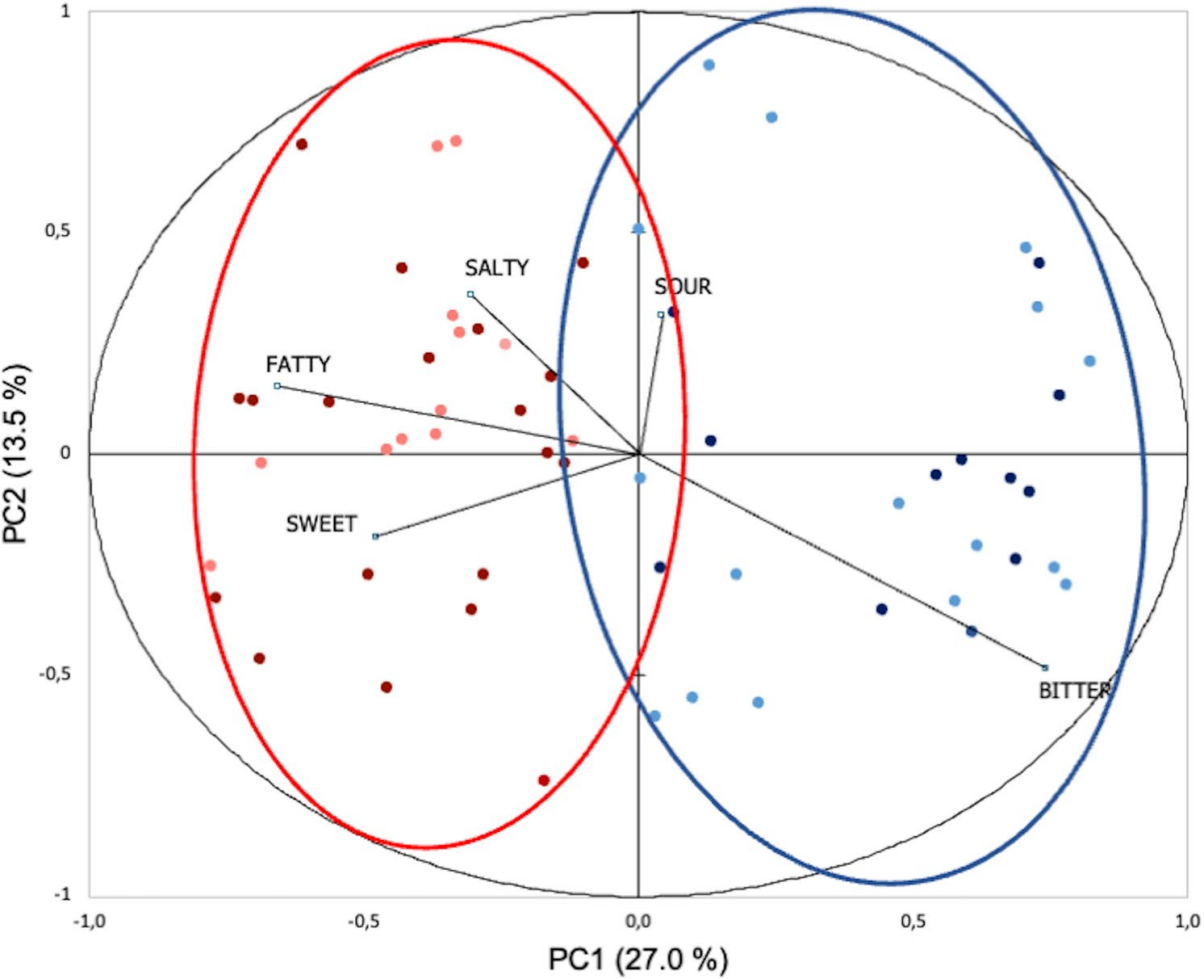
Loading from PC1–PC2 of the double-centered PCA on food liking resulting in two taste-liking clusters of Sweet-Salty-Fatty Likers (n = 31) and Bitter-Sour Likers (n = 28). Dot in dark colors (red and blue) depicted the children in the control group while dot in light colors (pink and light blue) depicted allergic children.

Foods characterized by sweet (mean liking = 5.2) and fatty (mean = 5.1) stimuli obtained the highest liking scores, while foods with a typical bitter (mean = 3.8) profile were the least liked. The sour (mean = 4.8) foods were scored above the foods characterized by bitterness. A double-centered PCA was used to map the children’s liking scores. The first two principal components accounted for 40.5% of the variability. Two clusters of Sweet-Salty-Fatty (52.5%, n = 31) and Bitter-Sour (47.5%, n = 28) Likers (Fig. 2) were defined on the basis of PCA loading scores.

No differences in terms of gender (χ^2^ = 0.14, p = 0.7) and presence/absence of allergy (χ^2^ = 2.8, p = 0.09) between the two clusters were observed. Bitter-Sour Likers are significantly older (t = 2.81, p < 0.01) than Sweet-Salty-Fatty Likers (mean age: 11.1 ± 2.0 and 9.6 ± 2.2, respectively).

To explore whether Liking clusters influence gustatory functions (i.e., sweet, sour, salty, bitter taste scores, TTS, and FP count) and personality traits (i.e., food neophobia), separate Mann–Whitney U tests and Student’s t-tests were performed. A significant Liking cluster effect on gustatory functions was found only for the Salty Taste Score (Mann–Whitney U value = 314.5, p = 0.05), with Bitter-Sour Likers who generally presented less difficulty identifying the salty taste quality than Sweet-Salty-Fatty Likers.No other significant differences were highlighted (Sweet Taste Score: Mann–Whitney U value = 3564.5, p = 0.17; Sour Taste Score: Mann–Whitney U value = 427.5, p = 0.92; Bitter Taste Score: Mann–Whitney U value = 343.5, p = 0.14; TTS: Mann–Whitney U value = 415.0, p = 0.77; FP count: t = − 1.2, p = 0.24).

### Taxonomic structure and ecological parameters of oral bacterial communities in allergic children

For investigating the composition of oral bacterial communities of allergic children (ALL), comparing it to that of sex- and age-matched controls (C), we extracted salivary DNA to produce and sequence 59 amplicons (29 from ALL patients, 30 from C), comprising the V3-V4 regions of the 16S rRNA gene. A total of 35,274 OTUs at the 97% homology level were obtained after the application of low count and low variance filters, clustered in 15 bacterial phyla, 28 classes, 57 orders, 119 families, and 257 genera. Supplementary Tables 1–5 reported the relative abundances of, respectively, phyla, classes, orders, families, and genera in individual samples.

**Figure 3:**
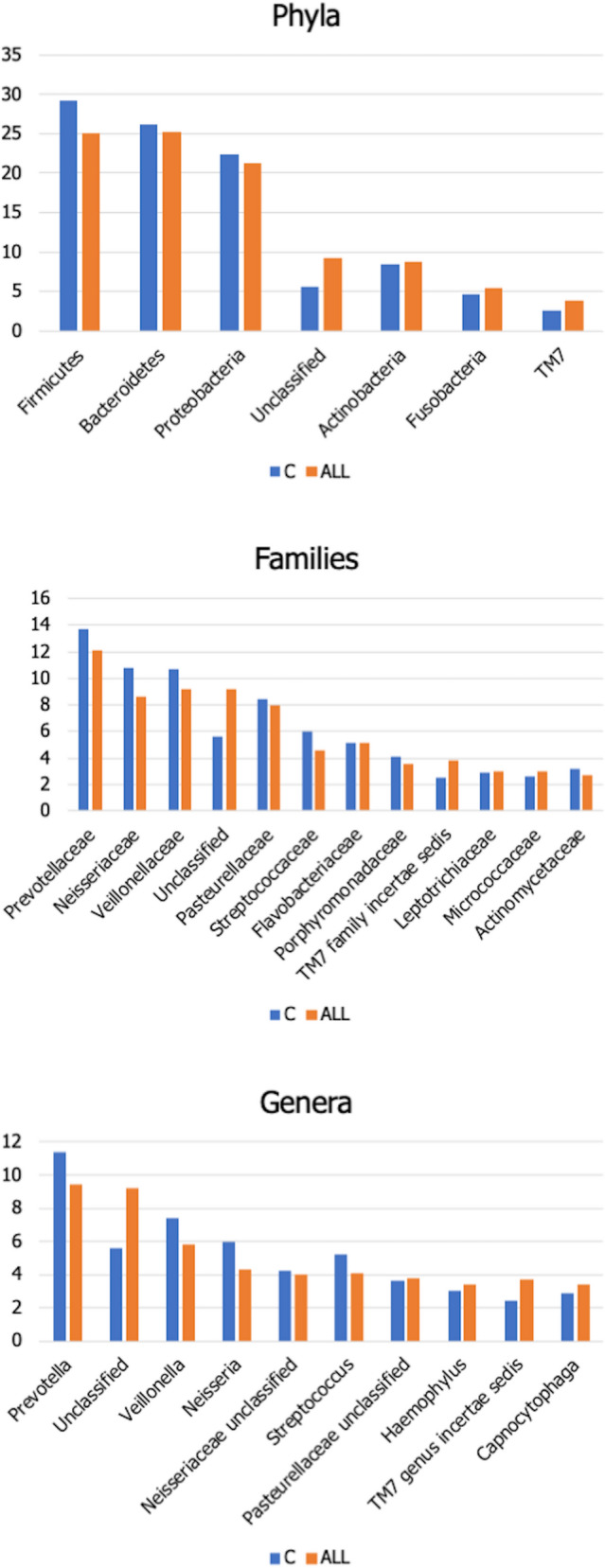
Taxonomic composition of the salivary microbiota in allergic (ALL) patients compared to sex- and age-matched healthy controls (C). Average relative abundance of the most represented phyla, families and genera identified in the two groups. Only taxa whose relative abundance is > 3% in at least one group are included.

The average relative abundance for the most represented phyla, families, and genera in ALL and C groups is shown in Fig. 3.

Concerning phyla, the two groups display comparable abundances of *Bacteroidetes*, *Proteobacteria,* and *Actinobacteria*. ALL patients appear characterized by the reduction of *Firmicutes* (25% *vs* 29.2% in controls) and the expansion of *Candidatus* Saccharibacteria (formerly known as TM7, belonging to the so-called Candidate Phyla Radiarion, CPR within the bacterial domain) and of a heterogeneous group of sequences representative of unclassified bacterial phyla. Concerning the taxonomic rank of families, the most abundant in both groups is *Prevotellaceae* (13.7% in C and 12.1 in ALL). Allergic children present a decrease in *Neisseriaceae* (class: *Betaproteobacteria*, 8.6% *vs* 10.8% in C), in the *Clostridiales* family of *Veillonellaceae* (9.2% *vs* 10.7 in C), and the *Lactobacillales* family of *Streptococcaceae* (4.6% *vs* 6% in C). On the other hand, they are characterized by the expansion of an uncharacterized bacterial family belonging to TM7 (3.8% vs 2.5%) and of unclassified bacterial families that, as for phyla, account for 9.2% of the diversity in patients (*vs* 5.6 in controls). Concerning genera, the picture reflects what is described above for families, with allergic patients characterized by a reduction of *Prevotella* (9.4% *vs* 11.4% in C), *Veillonella* (5.8% *vs* 7.4%), *Neisseria* (4.3% *vs* 6%), *Streptococcus* (4.1% *vs* 5.2%). On the contrary, in ALL patients, the expansion of unclassified taxa and TM7 is also seen at the genus level, with the same relative abundances observed for the higher taxonomic levels.

**Figure 4:**
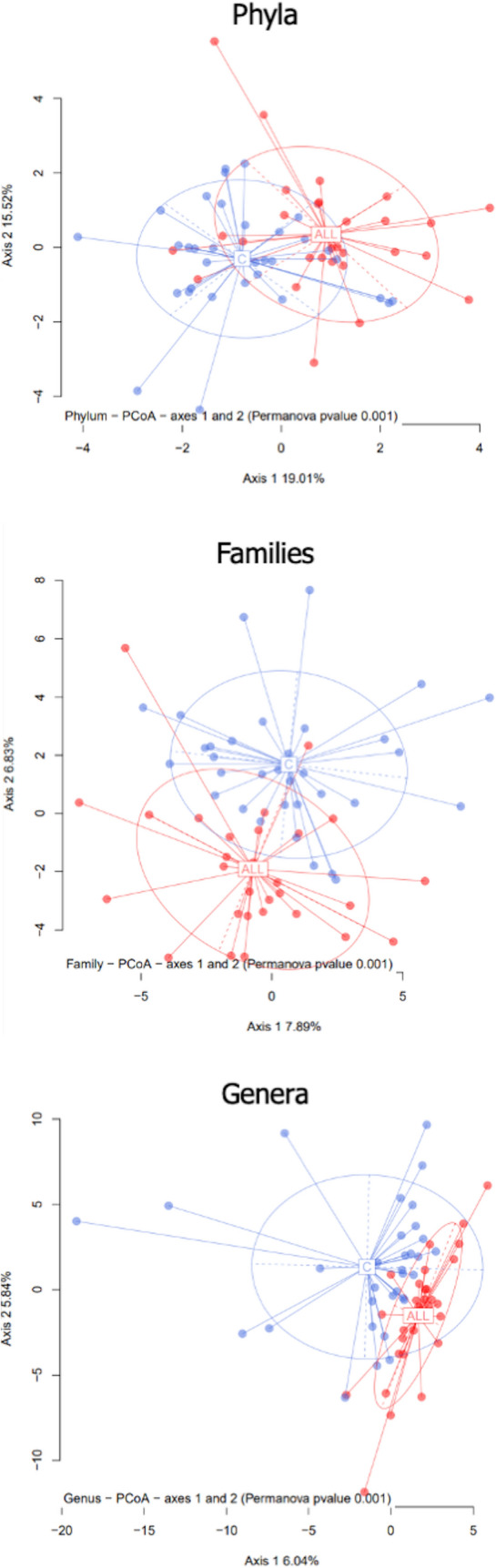
β-diversity analysis. The microbiota distances were visualized through Principal Coordinates Analysis (PcoA). The figure shows the taxonomic levels of phyla, families, and genera. The significance threshold (p-value) for the PERMANOVA was set < 0.001.

The comparison of within-sample diversity (α-diversity) indexes evidenced a trend of increasing values in patients that reached the statistical significance for the richness index Chao1 (average for ALL: 1881,48; average for C: 1490,2; Mann–Whitney U, p = 0.00033).β-diversity was computed to determine how bacterial taxa were differentially distributed in the two groups. A clear separation (verified by the PERMANOVA test) was observed between the two groups at all taxonomic rankings(all p-values < 0.001). Figure 4 shows the Principal Coordinates Analysis (PcoA) for the phyla, families, and genera taxonomic levels.

The differential taxa abundance between allergic patients and controls was also investigated using Mann–Whitney U and Kruskal–Wallis Rank Sum non-parametric tests. Table 2 lists the taxa displaying significantly different relative abundances in ALL and C groups.

**Table 2:** Taxa displaying significantly different relative abundances in ALL and C.

Taxon		Mean relative abundance		p-value
		C	ALL	
Phyla	*Firmicutes*	29.2	25	3.3 × 10^–6^
	Unclassified	5.6	9.2	5.3 × 10^–12^
Classes	Phylum: *Proteobacteria*			
	* Betaproteobacteria*	11.7	9.8	0.025
	Phylum: *Firmicutes*			
	* Bacilli*	14	10.6	4.9 × 10^–5^
	Unclassified	5.6	9.2	5.3 × 10^–12^
Orders	Phylum: *Proteobacteria*			
	* Neisseriales* (class*: Betaproteobacteria)*	10.8	8.8	0.011
	Phylum: *Firmicutes*			
	* Lactobacillales* (class*: Bacilli)*	14	10.6	4.9 × 10^–5^
	Unclassified	5.6	9.2	5.3 × 10^–12^
Families	Phylum: *Proteobacteria*			
	* Neisseriaceae* (order: *Neisseriales*)	10.8	8.6	0.011
	Phylum: *Firmicutes*			
	* Streptococcaceae* (order*: Lactobacillales)*	6	4.6	1.5 × 10^–4^
	Unclassified	5.6	9.2	5.3 × 10^–12^
Genera	Phylum: *Bacteroidetes*			
	* Prevotella* (family: *Prevotellaceae)*	11.4	9.4	0.01
	Phylum: *Proteobacteria*			
	* Neisseria* (family: *Enterobacteriaceae)*	6	4.3	7.1 × 10^–4^
	Phylum: Firmicutes			
	* Streptococcus* (family: *Streptococcaceae)*	5.2	4.1	3.7 × 10^–3^
	* Veillonella* (family: *Ruminococcaceae)*	7.4	5.8	0.023
	Unclassified	5.6	9.2	5.3 × 10^–12^

Wilcoxon–Mann–Whitney and Kruskal–Wallis Rank Sum non-parametric tests were applied, using a significance threshold (p-value) set to 0.05. For each taxon, the immediately higher taxonomic ranking and the phylum are indicated.

Taxa significantly depleted in ALL children comprehend the genus *Streptococcus* and its higher taxonomic rankings till the phylum *Firmicutes*; the genus *Neisseria* and the corresponding family (*Neisseriaceae*), order (*Neisseriales*) and class (*Betaproteobacteria*); the genera *Prevotella* and *Veillonella*. The most statistically significant difference between the two groups is represented by the enrichment, in ALL patients, of sequences not taxonomically resolved by the V3-V4 amplicon sequencing. This enrichment accompanies the ALL group at all taxonomic rankings, with the same relative abundances and an extremely significant p-value. The taxonomic identity of these sequences was further investigated by phylogenetically comparing them to the 16S sequences contained into the HOMD database (Human Oral Microbiota Database). Supplementary Fig. S1 shows the resulting phylogenetic tree, from which it emerges that these “unclassified sequences” form a monophyletic group and clusterize within *Candidatus* Gracilibacteria (formerly known as GNO2), another phylum within the CPR radiation. Some of these sequences were retrieved only from one of the two groups (C or ALL), while others from both, without a clear clustering.

## Discussion

Multifactorial causes underlie FA, which represents one of the most crucial causes of burden to children and their families. In this study, the hypothesis that differences exist in the taste perceptions of foods and in the microbial communities dwelling in the oral cavity between allergic and non-allergic children was investigated for the first time.

Food antigens and the immune system have the oral cavity as the first meeting place. Here, the functional roles of immunity and taste overlap, deciding what to ‘allow’ in and what to reject^34^. Thus, it is foreseeable that these systems may interact and influence each other. For example, it is known that taste cells and their signaling cascades are usually involved in auto- and hyper-immune diseases, over-expressing many genes associated with inflammation and innate immune response^35^. Moreover, in the past years, an activated inflammatory response has been correlated with impairments in taste perception in various chronic diseases, such as obesity^20,36^, diabetes^19,37^, and, more recently, the novel coronavirus infection^38–40^. Our results, which highlighted a reduced taste perception in children with food allergies, especially for bitter, sour, and salty tastes, support this assumption.

Taste perception was also evaluated by counting the number of fungiform papillae/cm^2^. Fungiform papillae are the gustatory anatomical structures containing taste buds and are mainly located in the anterior part of the tongue. The differential chemosensory perception among individuals has been previously linked to different fungiform papillae sizes and densities^41^. Our data supported the assumption that a direct correlation between fungiform papillae and taste perception might exist since allergic children presented a significantly lower papillae density besides impaired taste perception. This finding could be linked to the chronic low-grade inflammation reportedly associated with food allergy, which might impair the homeostasis of taste buds, decreasing their number both in the circumvallate and in the fungiform papillae, thus leading to alterations in taste. This hypothesis needs further mechanistic research and a better understanding of the interactions between taste and immune systems. However, even in the absence of studies dealing directly with food allergies, indirect support comes from previous research that associated respiratory allergies and hypersensitivity to impairments in olfactory and gustatory functions^42,43^.

Although a matter of debate in the last four decades, it is still unclear whether taste sensitivity measurements relate to food liking, and studies involving children and adolescents are still limited^44,45^. As expected, based on the list of 16 food items with specific taste profiles, children liked most foods, typically sweet, fatty, and salty^44,46^. We also evidenced some links between taste sensitivity and food liking, even though only related to salty taste. Indeed, Sweet-Salty-Fatty Likers seemed less sensitive to salty, presenting more difficulties in correctly identifying it than Bitter-Sour Likers. However, research about the effect of taste sensitivity on food liking is scarce and contradictory, both in adulthood and childhood, with some studies evidencing a relationship between some taste qualities (e.g.,^29,47,48^) and others not (e.g.,^44,49,50^). Possible explanations could lie in the difficulty of studying taste sensitivity in children and that other factors besides taste sensitivity could influence taste preferences (i.e., familiarity, environmental factors, role models of parents and siblings, and previous taste exposure)^51^.

With regard to eating habits, it has been reported that food neophobia significantly determines allergic children’s eating habits, influencing later in life their diet quality^14,15^. Owing to the allergy to a certain type of food, some individuals, especially children and adolescents, may continue not introducing the offending food even after having outgrown their food allergy, limiting one’s food preferences. With these premises, the present study compared the degree of food neophobia between allergic children and controls using a standardized scale validated on a large group of Italian children^52^. Our findings indicated that the allergic condition appeared to marginally influence the food neophobia attitude, with a higher, although not significant, neophobic attitude for the allergic group. This difference, even if represented only by a trend in our study, has been previously reported in a larger population of children diagnosed with one or more food allergies^13^, and in adults diagnosed with celiac disease^53^. However, due to the novelty of the issue of food neophobia in a clinical context, and the presence of controversial results (e.g.,^54^ arrived at opposite conclusions), it will be important to further evaluate this aspect in future studies.

In addition to sensory characteristics, our analysis of the oral milieu revealed that allergic patients displayed compositional shifts in oral microbiota profiles compared to non-allergic controls. The taxonomic picture retrieved in allergic patients is characterized by a significant down-representation of the phylum *Firmicutes* and, within it, of the genera *Veillonella* and *Streptococcus* (with all it is corresponding higher taxonomic rankings); of *Prevotella* spp. and of *Neisseria* spp. (and all the higher taxonomic rankings till the class *Betaproteobacteria*). Except for *Neisseria* (see below), our data agree with Ho and colleagues’ study^32^. They analyzed the oral milieu and the salivary microbiota in a cohort of children suffering from peanut allergy, recruited at the Mount Sinai Hospital, NY^32^. The fact that the same taxonomic changes characterize the oral microbiota of two cohorts of pediatric patients with different patterns of food allergies and with other geographic origins and eating habits (among the major "modifiers" of the microbiota community structure^55^) is noteworthy and makes it reasonable to hypothesize that such compositional shifts are strictly related to the state of food allergy itself. Ho and colleagues^32^, who also evaluated the oral metabolite and immunological profiles, linked the decreased abundances of *Prevotella* spp. and *Veillonella* spp. to reduced oral SCFA levels. Additionally, *Prevotella*oral abundance in children suffering from peanut allergy negatively correlated with the oral secretion of Th2 cytokines, such as interleukin 4 (IL-4), IL-5, and IL-13, and thus associated with the skew of oral immunity towards a Th2 milieu^32^. Notably, the same genera, together with *Streptococcus*, appeared depleted in the gut microbiota of children with food sensitization in early life^26,56,57^. The different results on *Neisseria* spp., whose oral prevalence resulted in an increase in allergic children from the Ho et al. work^32^ and decreased in our cohort, could be linked, in addition to the heterogeneous provenience of the populations under study, to the limited taxonomic resolution of 16S amplicon metagenomics, and the sequencing, in the two works, of different combinations of *Neisseria* species with different roles (e.g., as pathobionts or commensals).

As commonly noticed in works based on amplicon sequencing, a gap in the detection of microbial diversity emerges from our data. Indeed, a relevant portion of the community, accounting for 5.2% of bacterial lineages in controls and almost doubled in allergic patients (9.2%), consistently from phyla to genera, is represented by “unclassified bacteria”. This observation could explain the data on α-diversity which, contrary to expectations, appears to increase in the allergic group (Chao-1 richness estimator). This could result from the increase of a heterogeneous set of bacterial taxa for which taxonomic annotation is missing, presumably mostly low-abundance lineages, based on how the Chao-1 index is calculated^58^.

As to the taxonomic identity of these taxa, a systematic survey to evaluate commonly used 16S rRNA “universal” primers on gene sequences from over 6,000 assembled metagenomes has recently shown that > 70% of the bacterial clades systematically under-represented or missed in amplicon-based studies belong to Candidate Phyla Radiation or CPR^59^. CPR is a large monophyletic radiation of ultrasmall bacteria (> 73 phyla) believed to encompass > 25% of taxonomic diversity within the domain, with unique, and still poorly understood, genomic, metabolic and lifestyle features^60,61^. Indeed, a phylogenetic analysis confirmed this hypothesis and showed that sequences unresolved by the V3-V4 metagenomics, and doubled in ALL patients, clusterized within a CPR phylum, namely *Candidatus* Gracilibacteria (GNO2). This phylum, and others within CPR, as *Candidatus* Saccharibacteria (TM7), are stable components of the human microbiota, particularly abundant in the oral environment^62^. Interestingly, we have detected (and probably under-estimated) TM7 among the phyla, families, and genera with abundance > 3%, with a trend to increase in patients suffering from food allergies. This trend is in agreement with the available data on TM7, which, although scarce, are virtually the only available on the role of CPR bacteria in the ecology of the human microbiota^63,64^. TM7 bacteria increase in dysbiotic microbiomes and inflammatory environments, both in oral pathologies (e.g., periodontitis, gingivitis) and non-oral conditions (e.g., intestinal autoimmune disorders such as Inflammatory Bowel Disease, IBD), in which an increased TM7 diversity has also been reported^64^. The most immediate conclusion would be labeling TM7 as a pathogenic group. However, this idea has been recently ruled out by Chipashvili and colleagues^63^ who used mouse models to show that, instead, TM7 bacteria appear to attenuate oral inflammatory phenomena. This is largely dependent on their peculiar lifestyle as episymbionts of other bacteria, often pathobionts as *Actinomyces* spp., and their ability to modify traits of their hosts’ pathogenicity^63^. Finally, it has been shown that TM7 bacteria themselves present immunomodulatory features (e.g., they suppress Tumor Necrosis Factor-α, TNF-α expression in human macrophages)^64^. In light of these considerations, the fact that *Candidatus* Saccharibacteria (TM7) and other CPR bacteria, including *Candidatus* Gracilibacteria (GNO2), are abundant in the oral microbiota of children suffering from food allergy deserves further attention and research on their role in this pathological context.

As well, it would be of interest in future research the measurement of immune parameters in saliva or at a systemic level and their link with taste perception or microbiota composition, whose lack of assessment in the present study has to be mentioned as a limitation.

## Conclusions

In conclusion, interest in the sensory field with a specific focus on allergic diseases and food allergy is incipient, and mechanisms underlying taste performance are far from being understood. To date, this is the first study to put emphasis on the link between the sensory system and food allergy, from a one-health approach. Our findings indicate that the food allergic condition appears to impair taste perception, marginally influence the food neophobia attitude, and is linked to compositional shifts in oral microbiota. However, oral microbiota’s role in food allergy remains largely unexplored, and some groups of bacteria (i.e., GNO2, TM7 and possibly other lineages within CPR) certainly deserve further attention and research. Finally, given the contribution of taxonomically unresolved taxa to the differential taxonomic picture of food allergy patients *vs* controls, a more realistic 16S metagenomic picture of this, as well as of probably several other pathological conditions, would certainly require to use sequencing protocols able to include those portions of the bacterial community lost using standard workflows on the Illumina platform, e.g., exploiting more specific primer pairs and/or sequencing whole 16S amplicons.

Appropriate investigation of these factors and their inter-relationships will require collaborative efforts between multidisciplinary teams and will likely open exciting new approaches to therapeutic interventions.

## Materials and methods

### Participants

The participants were recruited at the Pediatric Clinic of the V. Buzzi Children’s Hospital (Milan, Italy). Allergic children were regularly followed at the clinic since their diagnosis of food allergy. The inclusion criteria were: diagnosis of IgE-mediated food allergy (presence of positive skin prick tests and/or specific IgE), confirmed by an oral food challenge, except in case of a clinical history of anaphylaxis, and allergen avoidance diet since at least 6 months, age range 6–14 years old, and Caucasian ethnic group. We excluded patients treated with medications affecting smell or taste and antibiotics within 2 months before the study. A group of healthy sex- and aged-matched controls were recruited as controls from other clinic departments, using the same exclusion criteria. A matching method was used to precisely match cases with non-allergic controls on the basis of sex (same) and age (within 1 year), thus preventing any bias in analyzing the FP count as physiological sensitivity marker, as well as a cognitive bias in taste recognition responses. Using data from a pilot study and previous research^20^, to detect a difference ≥ 1 in taste recognition ability between the two groups, a total sample size of n = 54 subjects (27 in each group) will allow testing for strong effect sizes of d = 0.8 (std. dev. = 2.5, α = 0.05, β = 0.8) as calculated with G-power.

### Ethics statement

The study was conducted according to the Declaration of Helsinki, and all methods followed the relevant guidelines and regulations. The Ethical Committee of ASST-Fatebenefratelli-Sacco (Ref n. 2021/ST/041) approved the study. The privacy rights of subjects have been observed. The parents of the children involved signed an informed consent form to allow their child to participate.

### General procedure

Participants in a fasted state were subjected to 4 successive tasks. Task 1: an interview with parents and screening by the medical team. Task 2: a collection of the oral samplings of saliva. Task 3: the taste sensitivity assessment. Task 4: fill in a food neophobia questionnaire and complete a food liking questionnaire.

### Saliva collection and amplicon production

#### Collection of saliva and DNA extraction

Children in a fasted state were asked to accumulate unstimulated saliva in the floor of the mouth and then spit it into a graduated and sterile plastic tube. The participants should provide the unstimulated whole saliva samples in a time span not exceeding 10 min (min). Then, the sample was immediately frozen. The QIAamp DNA Blood Mini Kit, Qiagen (Hilden, DE), was used to extract DNA from 1 ml saliva, following the manufacturer’s protocol^20^. The DNA concentration of extracted samples was assessed fluorometrically^20^.

#### PCR production of 16S rRNA amplicons (V3–V4 regions) and sequencing

The V3–V4 hypervariable regions of the prokaryotic 16S rRNA gene were targeted for amplicon production. Amplicon production and sequencing on an Illumina MiSeq platform were performed as previously reported in^20^.

### Taste sensitivity assessment

#### Taste recognition ability

Taste sensitivity assessment was performed applying the ‘Taste Strips’ method^65^, using prefabricated filter papers (Taste Strips, Burghart, Wedel, Germany) impregnated with four increasing concentrations of sweet, sour, salty, and bitter taste qualities, plus two tasteless strips. The detailed protocol used is fully described in^19,20^.

#### Fungiform Papillae (FP) count

The procedure used to assess FP count was fully described in^20^. In brief, the FP was performed by counting the number of these structures inside three 6 mm-diameter circles virtually drawn on the photo of the subject’s anterior part of the tongue, using Adobe Photoshop software (Adobe Systems Incorporated, San Jose, California)^66^. The count inside each circle was repeated twice by two independent examiners (blinded to food allergy status) following the Denver Papillae Protocol^67^. Then, the mean of the two counts was calculated.

### Food neophobia

The assessment of children’s food neophobia was performed through Italian Children Food Neophobia Scale (ICFNS)^52^. Children were asked to express the level of agreement/disagreement for eight statements, using a 5-point facial expression scale (‘very false to me’ – ‘very true for me’). The individual Food Neophobia (FN) score was computed as the sum of given ratings, thus, the scores theoretically ranged from 8 to 40, with higher scores reflecting higher FN levels.

### Liking questionnaire

A food-liking questionnaire adapted from^19^ was completed by children. The questionnaire consisted of 16 different food items characterized by specific taste profiles (i.e., four basic tastes and fattiness) defined in a previous study^68^. Liking was evaluated on a 7-point facial hedonic scale (1 ‘super bad’ – 7 ‘super good’).

### Data analysis

Data were presented as means ± SD for normally distributed variables or median ± IQR for variables that did not fit normality assumptions. Variables of interest were checked for normality and verified according to the Shapiro–Wilk test (W range: 0.66–0.91, p < 0.001).

To determine whether a between-group effect occurs for age and BMI, the independent samples t-test was used. Similarly, a χ^2^ test was used to evaluate differences in sex proportions between the two groups. These analyses were carried out to verify the sex- and age match between allergic and controls.

Group-related (allergic *vs* controls) and sex-related differences in gustatory ability, expressed as single taste scores (i.e., sweet, sour, salty, and bitter scores, each ranging from 0 to 4) and the cumulative one (total taste score, TTS; range 0–16), were statistically evaluated with separated Mann–Whitney U tests. Dunn’s test with Bonferroni correction for multiple comparisons was used as a post hoc test when statistically significant differences were observed. FP count was statistically evaluated with a separate Student’s t-test.

To assess whether group-related (allergic *vs* controls) and sex-related differences were associated with Food neophobia, FN scores were submitted to 2-way ANOVA considering Group (allergic *vs* controls), sex, and their interaction as factors.

Children’s food liking was mapped through a principal component analysis (PCA), considering the 16 food items as rows and children’s liking scores as columns. The food-liking scores were double-centered before the analysis^69^. Food’s taste quality was coded as binary variables (1 = present, 0 = not present) for each of the four basic tastes and fattiness and entered as supplementary variables. Based on PC1 and PC2 loadings, children were clustered into two liking groups. Independent samples t-tests were used to determine whether a between-group effect occurs for age and BMI. Similarly, χ^2^ tests were used to evaluate differences in sex and allergic/control subjects’ proportions between the two groups. To explore whether Liking clusters influence gustatory functions (i.e., sweet, sour, salty, bitter taste scores, TTS, and FP count) and personality traits (i.e., food neophobia), separated Mann–Whitney U tests and Student’s t-test were performed where appropriate. A p-value of 0.05 was considered significant. The SPSS 27.1 (IBM, Armonk, New York) and XLSTAT Sensory package (version 2021.4.1, Addinsoft, Boston, MA, USA) were used for the data analysis.

The bioinformatics analysis of microbiota sequencing data was based on the Mothur pipeline^70^. Raw FASTQ files were quality-filtered using Trimmomatic^71^ and high-quality reads were analysed following the SOP Mothur procedure, as described in^20^. The main ecological indexes of within-sample, α-diversity (Shannon, Chao, inverse Simpson) were computed using Mothur. Diversity in composition among samples (β-diversity) was evaluated by plotting the relative heatmap using the function heatmap.2 of the Gplots R library^72^ and the relative Principal Component Analysis (PCoA) using the R library Ade4^73^. The Permanova analysis was performed by using the R library Vegan^74^. Microbial profiles of patients and controls were finally compared to evidence statistically significant differences in bacterial composition and taxa abundances, using Mann–Whitney U test with a significance threshold (p-value) set to 0.05.

After metagenomic analysis, the representative sequence of the most abundant OTUs “unclassified” by the Mothur pipeline, and counted at least 20 times in at least one sample, were selected. These sequences were Blastn-searched against the HOMD 16S database (https://www.homd.org/) and, for each, the 50 more similar sequences were retrieved. The obtained dataset, including the selected OTUs and HOMD entries, was aligned using Muscle^75^ and subjected to Maxumum Likelihood (ML) phylogenetic analysis using RAxML8^76^ with 100 pseudobootstraps.

## Supplementary Information

Supplementary Figure S1.

Supplementary Table 1.

Supplementary Table 2.

Supplementary Table 3.

Supplementary Table 4.

Supplementary Table 5.

## Data Availability

The datasets generated during and/or analyzed during the current study are available from the corresponding author upon reasonable request.
