# Supplementary material for: Alteration of taste perception, food neophobia and oral microbiota composition in children with food allergy
Source: Sci Rep. 2023 Apr 28;13:7010. doi: 10.1038/s41598-023-34113-y (PMC10147366; doi:10.1038/s41598-023-34113-y)
Supplement: Supplementary file 1 — Supplementary Figure S1. [file 41598_2023_34113_MOESM1_ESM.pdf]

Alteration of taste perception, food neophobia and oral microbiota composition in children with food allergy

Enza D'Auria[1], Camilla Cattaneo[2]*, Simona Panelli[3], Carlotta Pozzi[1], Miriam Acunzo[1], Stella Papaleo[3], Francesco Comandatore[3], Chiara Mameli[1], Claudio Bandi[4], Gianvincenzo Zuccotti[1,3], Ella Pagliarini[2]

[1]Department of Pediatrics, Buzzi Children's Hospital, University of Milan, Milan, 20154, Italy

[2]Sensory & Consumer Science Lab (SCS_Lab), Department of Food, Environmental and Nutritional Sciences, University of Milan, Milan, 20133, Italy

[3]Pediatric Clinical Research Center "Invernizzi", Department of Biomedical and Clinical Sciences, University of Milan, Milan, 20157, Italy

[4]Pediatric Clinical Research Center "Invernizzi", Department of Biosciences, University of Milan, Milan, 20157, Italy

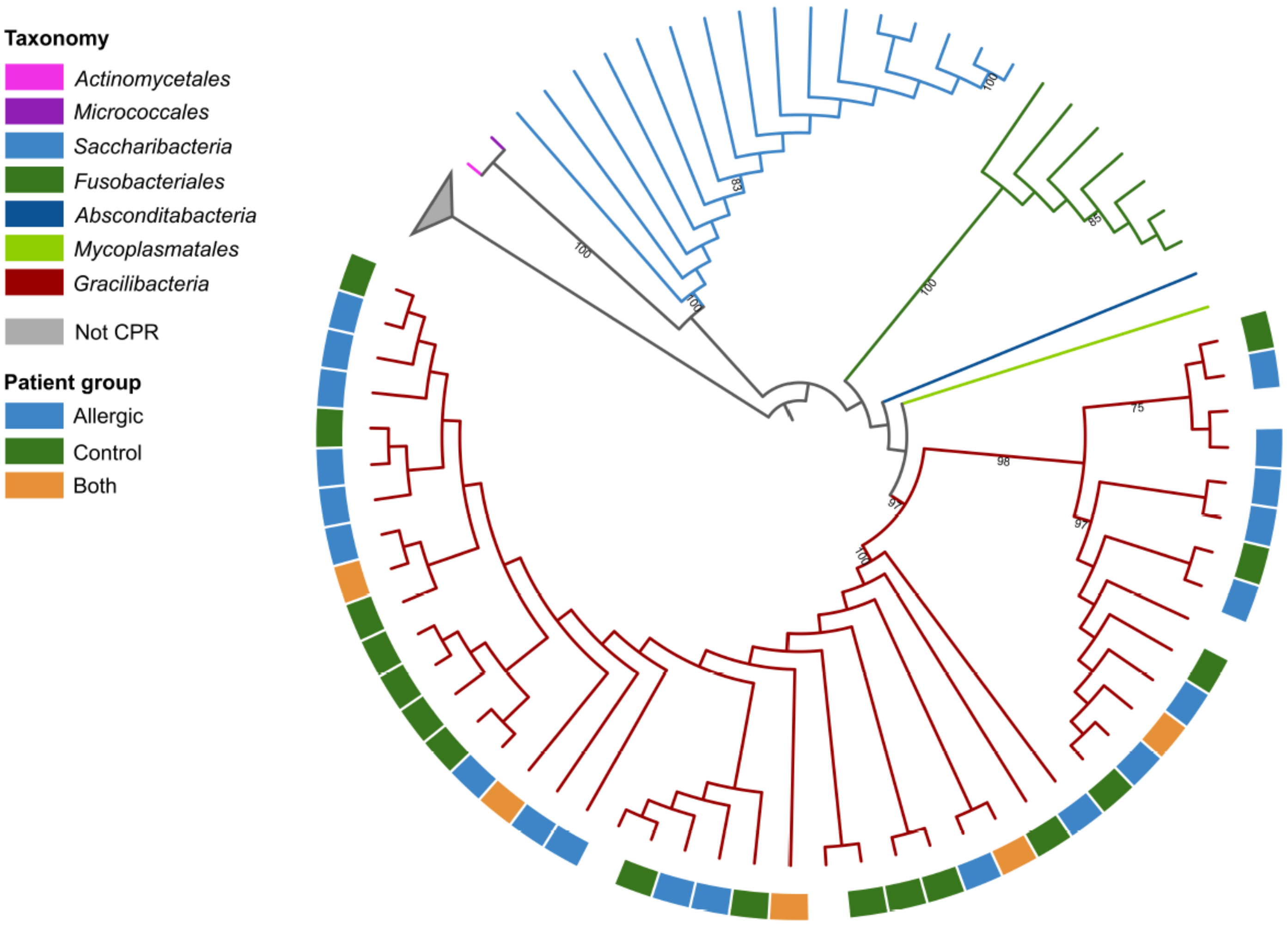

Supplementary Figure S1. Phylogenetic analysis of «unclassified sequences». The most abundant sequences among those unresolved by the V3-V4 metagenomics (those that could be counted at least 20 times in at least one sample) were selected and Blastn-searched against the database HOMD (Human Oral Microbiome Database). The first 50 hits for each sequence were passed to RAxML8 software and used for Maximum Likelihood tree construction (bootstrap support values >75% are shown). Tree branches are colored following their taxonomy (see legend).
Our selected sequences (see above) are signalled on the tree by colored rectangles. Blue, green and yellow rectangles refer to sequences found in ALL, C and both (ALL+C) groups, respectively.
